# Effectiveness of a Web-Based Intervention for Preventing Substance Use in Young Adults in Taiwan: Quasi-Experimental Study

**DOI:** 10.2196/40157

**Published:** 2023-08-14

**Authors:** Yen-Jung Chang, Jhong-Lin Chen

**Affiliations:** 1 Department of Health Promotion and Health Education National Taiwan Normal University Taipei Taiwan

**Keywords:** controlled substances, health education, illicit drugs, prevention, substance use, web-based intervention, young adults

## Abstract

**Background:**

Substance use has been one of the most alarming public health problems worldwide, particularly among younger generations.

**Objective:**

This study evaluated the effectiveness of a web-based substance use prevention intervention targeted at adults aged 20-29 years.

**Methods:**

The intervention materials comprised 5 sets of infographics and 1 animation, all of which focused on mixed themes: (1) the concept of substance use and its harmful effects on health; (2) misinformation regarding new psychoactive substances; (3) regulation of illicit drugs, particularly marijuana; (4) the brain disease model of addiction; (5) critical thinking skills that improve health literacy; and (6) decision-making and communication skills that help people refuse illegal drugs. The study assigned eligible participants into experimental and control groups on the basis of the parity of their participant numbers. These participants completed web-based baseline and follow-up questionnaires that assessed their knowledge, behavioral intention, self-efficacy, and life skills related to substance use prevention. Knowledge was assessed using 8 questions concerning understanding of substance use harms and the regulation of illicit drugs. Behavioral intention and self-efficacy were assessed using 5-point Likert-type scales. Participants’ ability to apply life skills to avoid substance use was assessed using 3 testing scenarios regarding substance use. The study used generalized estimating equations to examine the intervention’s effectiveness.

**Results:**

A total of 1065 participants (539 control and 526 experimental) completed the intervention and questionnaires in 2019. The average ages of the experimental and control groups were 25.68 (SD 2.71) and 25.66 (SD 2.69) years, respectively. The study observed no significant differences in the demographic variables between the 2 groups. The results of the generalized estimating equation analyses indicated that the intervention significantly improved participants’ knowledge (*P*<.001), behavioral intention (*P*<.001), and self-efficacy (*P*<.001) but not their life skills (*P*=.61) related to substance use prevention. Participants in the experimental group responded to a satisfaction survey with positive feedback on the intervention.

**Conclusions:**

The web-based intervention was effective in improving participants’ knowledge, behavioral intention, and self-efficacy concerning substance use prevention. The findings support continued efforts to use web-based interventions to prevent substance use among young adults.

## Introduction

According to the 2020 World Drug Report, drug use has increased rapidly worldwide over the past 2 decades, particularly among younger generations [1]. The 2020 Annual Report of Drug Use Statistics in Taiwan reported that over the previous 5 years, approximately 44.7% of first-time illicit drug users were aged between 20 and 29 years, and the main drugs of choice were methamphetamine, ketamine, and marijuana [2], suggesting that the planning of relevant interventions for young adults is urgent. Although substance use problems are alarming among younger populations, these individuals might not regularly receive prevention education in their communities, as most programs are implemented in schools [3-5].

The internet has given rise to a new platform for substance use prevention education. Web-based interventions have the potential to increase participants’ understanding of illegal drugs and their risks with comparable effectiveness to that of traditional classroom lectures. Furthermore, the application of web-based interventions may assist school health education and increase students’ willingness to learn [6]. The effectiveness of these web-based interventions in reducing alcohol or marijuana use in adolescents has also been reported [7-9].

Studies have attempted to leverage web-based interventions for substance use prevention education and shown mixed evidence [10,11]. Theory-based design of health intervention contents is recommended to improve the quality and increase the impact of digital interventions [12]. Interventions should not only transmit knowledge but also emphasize the cultivation of attitudes and skills to trigger behavior change effectively. This study, applying behavior theory–based content design, implemented and evaluated a web-based substance use prevention intervention targeted at young adults aged 20-29 years in Taiwan in 2019. The objectives of the study were to examine whether the intervention could improve participants’ knowledge and self-efficacy of substance use prevention and develop their abilities to apply life skills in substance use–related scenarios.

## Methods

### Research Participants

The study selected research participants from young adult internet users aged 20-29 years in Taiwan. The internet use rate reached 100% among a representative sample of Taiwanese people aged 20-29 in 2020 [13]. The study recruited a total of 1200 participants on the internet at baseline from August to October 2019, and 1065 completed the intervention and the follow-up assessment (526 in the experimental group and 539 in the control group). Informed consent was obtained from all participants.

### Ethics Approval

The study procedure and materials were approved by the institutional review board of the National Taiwan Normal University (#201903HM009).

### Research Design

The study based the design of the intervention materials on a literature review, needs assessment, behavioral theories, and meetings with experts. The needs assessment stage involved quantitative and qualitative methods. The quantitative component involved a web-based survey of the learning needs and interests related to substance use prevention of 357 participants aged 20-29 years. The qualitative component involved the implementation of in-depth interviews with young substance users and health care professionals involved in addiction treatment to understand their motives, experiences, and reflections of substance use as references for compiling the intervention materials.

The study primarily based the intervention content design on need assessment results and behavior theories, including the health belief model [14], theory of planned behavior [15,16], and social cognitive theory [17]. This design aimed to increase young individuals’ awareness of the health harms from substance use and the benefits of avoiding substance use, enhance individuals’ self-efficacy to reject substance use, help individuals form an unfavorable evaluation of substance use outcomes, and facilitate individuals’ healthy behavior regulation through observational learning from case examples. The intervention materials included 5 sets of web-based infographics and 1 animation. Each set of infographics had a specific objective and consisted of 10-12 pictures with brief narratives. The following themes were mixed and presented in the infographic sets and animation: (1) understanding substance use and its harmful effects on health; (2) misinformation regarding new psychoactive substances; (3) laws against using illicit drugs, particularly marijuana; (4) the brain disease model of addiction; (5) critical thinking skills that help improve health literacy; and (6) decision-making and communication skills that help the refusal of illegal drugs. For example, the animation adopted themes (1) and (6) and aimed to encourage participants to evaluate the negative consequences of unhealthy substance use and make a healthy decision to refuse substance use. A set of infographics conveyed themes (2) and (5) to teach participants how to verify the misinformation on the internet regarding new psychoactive substances. The learning objectives of the other sets of infographics included recognizing risky substance use behaviors in daily life, reducing the intention of using addictive substances through knowing and analyzing the physical and social harms of addictive substance use, and preventing the negative effects of addictive substance use on brain functions.

This study used a quasi-experimental design, given that the implementation of random assignment was not feasible at baseline under resource constraints. All intervention activities were conducted on the web-based platform, which displayed the intervention materials and questionnaires. The study divided all eligible participants, after their completion of the baseline assessment, into experimental and control groups on the basis of the parity of their participant numbers, which they were assigned upon accessing the platform. The study guided participants in the experimental group to review all of the infographics and watch the animation, whereas the study asked those in the control group to watch a set of videos about reproductive health education. The study asked participants in both groups to complete a follow-up evaluation questionnaire immediately after participating in the preceding activities.

### Measurement

Participants’ knowledge on substance use prevention was assessed using 8 questions concerning the understanding of substance use harms (eg, the health effects of using methamphetamine) and the regulation of illicit drugs (eg, the legal consequences of purchasing marijuana through the internet). The study calculated the scores referring to the correct responses for each participant, with a higher score indicating a greater understanding. Behavioral intention, the participants’ self-rated likelihood of avoiding substance use, was assessed using a 5-point Likert-type scale comprising 4 items, such as “Please rate the likelihood of you refusing substance use if you are invited by friends to try using an additive substance.” Higher scores represented a stronger behavioral intention to avoid substance use. The self-efficacy measure also used a 5-point Likert-type scale with 4 items, such as “Please rate the level of confidence toward your ability to verify the online misinformation about using addictive substances.” Higher scores indicated greater confidence in the ability to resist substance use. Finally, the study assessed participants’ ability to apply life skills to avoid substance use, using 3 testing scenarios (eg, “How would you respond to an invitation from colleagues to try addictive substances?”). The higher scores of correct responses indicated a better ability to apply life skills to prevent substance use in daily life. These measurements were developed along with the intervention content design on need assessment results and behavior theories.

### Statistical Analysis

The study used SAS (version 9.4; SAS Institute Inc) to analyze the data. Characteristics and baseline data of 2 groups of participants were compared using an independent samples *t* test. The study used generalized estimating equations (GEEs) to examine whether the web-based intervention had improved participants’ knowledge, behavioral intentions, self-efficacy, and life skills concerning substance use prevention. The GEE method can be used to analyze repeated measures with non-normal response variables, and it can take into account the correlation of within-subject data.

## Results

### Characteristics of the Study Participants

The average ages of all 1065 participants, the experimental group, and the control group participants were 25.67, 25.68, and 25.66 years, respectively. Both the experimental and control groups had a higher proportion of women than men (56.8% and 52.7%, respectively). The study observed no significant differences in the demographic variables between the 2 groups, as displayed in Table 1.

**Table 1:** Participant demographics and descriptive statistics regarding knowledge, behavioral intentions, self-efficacy, and life skills (N=1065).

			Control group (n=539)	Experimental group (n=526)
**Participant demographics**				
	Age (years), mean (SD)		25.66 (2.69)	25.68 (2.71)
	**Gender, n (%)**			
		Man	255 (47.3)	227 (43.2)
		Woman	284 (52.7)	299 (56.8)
**Descriptive statistics of intervention outcomes**				
	**Knowledge (range: 0-8), mean (SD)**			
		Baseline^a^	5.84 (1.36)	5.60 (1.52)
		Follow-up^b^	6.08 (1.36)	6.32 (1.52)
	**Behavioral intention (range: 1-5), mean (SD)**			
		Baseline	4.62 (0.54)	4.56 (0.62)
		Follow-up^a^	4.74 (0.50)	4.79 (0.45)
	**Self-efficacy (range: 1-5), mean (SD)**			
		Baseline	4.70 (0.48)	4.65 (0.53)
		Follow-up^b^	4.76 (0.44)	4.83 (0.42)
	**Life skills (range: 0-3), mean (SD)**			
		Baseline	2.46 (0.60)	2.40 (0.69)
		Follow-up	2.49 (0.57)	2.46 (0.57)

^a^*P*<.05.

^b^*P*<.01.

### Knowledge of Substance Use Prevention

At baseline, the average scores of the knowledge scale in the control and experimental groups were 5.84 and 5.60, respectively. The rates of correct answers in the control and experimental groups were 73% and 70%, respectively. An independent samples *t* test indicated that the control group scored significantly higher (*P*<.05). In the follow-up test, the average scores of the control and experimental groups on the questions in the same section were 6.08 and 6.32, respectively. The study observed a significant difference between the 2 groups (*P*<.01), with the experimental group scoring higher (see Table 1). The GEE analysis indicated a significant interaction between the intervention group and time (*P*<.001; see Table 2), suggesting that the intervention materials were effective in enhancing participants’ knowledge of substance use prevention.

**Table 2:** Generalized estimating equation analysis of the effectiveness of the intervention for improving the knowledge of substance use prevention (N=1065). Model: Y=β0+β1(group)+β2(time)+β3(group×time).

Parameter	Estimate（β）	SE	*P* value	95% CI
Intercept	.731	0.0074	<.001	0.716 to 0.745
Group (experimental vs control^a^)	–.024	0.0112	.03	–0.046 to –0.002
Time (follow-up vs baseline^a^)	.030	0.0046	<.001	0.021 to 0.039
Group×time	.053	0.0082	<.001	0.037 to 0.069

^a^Reference group.

### Behavioral Intention Regarding Substance Use Prevention

In the baseline test, no significant difference (*P*>.05) was found between the control (4.62) and experimental (4.56) group scores for behavioral intention. In the follow-up test, the mean scores of the control and experimental groups on the questions in the same section were 4.47 and 4.79, respectively, and the results of the *t* test revealed a significant difference (*P*<.01) between the 2 groups (Table 1). The GEE analysis indicated that the interaction term of group and time was significant (*P*<.001; Table 3), suggesting that the intervention materials were effective in improving participants’ behavioral intention to avoid substance use.

**Table 3:** Generalized estimating equation analysis of the effectiveness of the intervention for improving behavioral intention in avoiding substance use (N=1065). Model: Y=β0+β1(group)+β2(time)+β3(group×time).

Parameter	Estimate（β）	SE	*P* value	95% CI
Intercept	4.625	0.0231	<.001	4.580 to 4.670
Group (experimental vs control^a^)	–.065	0.0356	.07	–0.135 to 0.005
Time (follow-up vs baseline^a^)	.112	0.0185	<.001	0.075 to 0.148
Group×time	.123	0.0292	<.001	0.066 to 0.18

^a^Reference group.

### Self-Efficacy in Resisting Substance Use

In the baseline test, the study observed no significant differences (*P*>.05) between the control (4.70) and experimental (4.65) groups in their average self-efficacy scores for resisting substance use. In the follow-up test, the experimental group (4.74) outperformed the control group (4.79; *P*<.01; see Table 1). The results of the GEE analysis confirmed the significant interaction of group and time (*P*<.001; Table 4), suggesting that the intervention materials were effective in improving self-efficacy in resisting substance use.

**Table 4:** Generalized estimating equation analysis of the effectiveness of the intervention for improving self-efficacy in preventing substance use (N=1065). Model: Y=β0+β1(group)+β2(time)+β3(group×time).

Parameter	Estimate（β）	SE	*P* value	95% CI
Intercept	4.697	0.0209	<.001	4.656 to 4.738
Group (experimental vs control^a^)	–.044	0.0310	.16	–0.104 to 0.017
Time (follow-up vs baseline^a^)	.060	0.0164	<.001	0.028 to 0.092
Group×time	.113	0.0239	<.001	0.066 to 0.16

^a^Reference group.

### Life Skills to Prevent Substance Use

The average scores of the control and experimental groups on the questions regarding the life skills scenarios were 2.46 and 2.40, respectively, at baseline, and 2.49 and 2.46 on the follow-up test, respectively. There was no significant difference in the scores between the 2 groups at baseline or in the follow-up (Table 1). According to the results of the GEE analysis, the intervention materials did not significantly improve participants’ life skills with respect to resisting substance use (*P*=.61; see Table 5).

**Table 5:** Generalized estimating equation analysis of the effectiveness of the intervention for improving life skills for preventing substance use (N=1065). Model: Y=β0+β1(group)+β2(time)+β3(group×time).

Parameter	Estimate（β）	SE	*P* value	95% CI
Intercept	.825	0.0084	<.001	0.808 to 0.841
Group (experimental versus control^a^)	–.022	0.0132	.09	–0.048 to 0.004
Time (follow-up versus baseline^a^)	.011	0.0069	.13	–0.003 to 0.024
Group×time	.006	0.0111	.61	–0.016 to 0.027

^a^Reference group.

### Experimental Group’s Satisfaction With the Intervention

The experimental group completed an additional survey consisting of 4 questions regarding their satisfaction with the intervention materials. The questions used a 5-point Likert-type scale, with higher scores indicating a more positive reception of the intervention materials. The average scores for each question are presented in Table 6. All scores fell between 4 (satisfied, helpful, and recommended) and 5 (very satisfied, very helpful, and highly recommended).

**Table 6:** Descriptive statistics regarding experimental group’s satisfaction with intervention materials (N=526). Questions scored between 1 and 5; higher scores implied greater satisfaction.

Question	Mean (SD)
1. In general, how satisfied were you with the infographics?	4.31 (1.15)
2. In general, how satisfied were you with the animation?	4.27 (1.15)
3. Do you think that the intervention materials were helpful in preventing you from using substances in the future?	4.33 (1.14)
4. Are you willing to recommend the intervention materials to your family and friends?	4.19 (1.23)

## Discussion

### Principal Findings and Comparison to Previous Work

The study results indicated that the web-based intervention significantly increased participants’ knowledge and self-efficacy in substance use prevention. This intervention might also enhance participants’ behavioral intention of avoiding substance use even when they have been exposed to risky substance use situations. According to the Theory of Planned Behavior, behavioral intention is a significant predictor of behavioral performance [15,16]. Although this study did not observe actual behavior changes after the intervention, we expected that the stronger the intention to reject friends’ invitation to use substances, the more likely the action of rejecting substance use will be performed. However, the intervention materials did not improve participants’ life skills with regard to resisting substance use. During the development of the intervention, both the literature review and expert committee highlighted the importance of acquiring life skills to prevent substance use; however, the results of this study indicated that short-term exposure to web-based materials, such as infographics and animations, is likely ineffective for improving the targeted skills. In addition to being aware of the concept and importance of life skills, study participants might also need opportunities to practice. Future learning materials to build life skills might consider simulation practice. An example of this can be seen in a previous Taiwanese study in which a multimedia tool with games was developed to encourage the interactive practice of such skills among students, with the results suggesting that the intervention was effective [18]. Therefore, we suggest that interventions targeting life skills for resisting substance use incorporate materials in forms other than infographics or animations and instead provide practical opportunities through interactive approaches that guide learners in applying their acquired skills in real life.

Participants in the experimental group responded with highly positive feedback on the intervention content and form. Participants also made constructive suggestions for further promotion (not shown in tables), such as featuring internet celebrities, providing additional animations, and displaying intervention messages on public transportation digital displays and screens. Similar strategies were deployed in cancer awareness campaigns targeting young women, and the campaigns made a positive impact as an effective public health communication initiative [19]. Future web-based health interventions could benefit from these feedback and suggestions and capture the attention of the younger generation. In previous literature, substance use prevention has primarily focused on school-aged youth in family and school settings [3,4,20]. Adolescents and young adults might have different learning needs and interests. Given the reported age of first-time drug users in Taiwan, we recommend more prevention activities targeting emerging adults, with a focus on a web-based delivery approach.

### Limitations

Several limitations in this study should be mentioned. First, the participants took the follow-up test immediately after the intervention; thus, the long-term outcome or lag effect was not observed. Second, for the self-report questionnaires, using a single format of Likert-type items might have resulted in eliciting routine response patterns and socially desirable responses, which might have biased the results. Questions concerning substance use might be considered sensitive information by some participants. The study made the questionnaires anonymous to encourage truthful answers; however, social desirability bias could still have affected the responses. Third, factors affecting substance use behaviors are complicated, and social or cultural factors were not comprehensively addressed in this intervention, although these factors may also shape substance use behaviors. Finally, we recruited the study participants through convenience sampling, which may not be representative of the entire young adult population (aged 20-29 years) in Taiwan.

### Conclusions and Future Directions

In conclusion, this web-based substance use prevention intervention has the potential to enhance the knowledge, self-efficacy, and behavioral intention of young adults concerning substance use prevention. The high internet use rate among young people in Taiwan can facilitate the adoption of the web-based intervention. Web-based intervention promotion and diverse topics of materials (eg, materials that target various groups or provide coping strategies for various risk scenarios) should be developed further to promote substance use prevention in young people.

## Abbreviations

GEE: generalized estimating equation

## Data Availability

The data presented in this study are available upon reasonable request from the corresponding author.
